# Drug substitutes − Insights on benefits, risks, detection methods, and management strategies: a systematic review

**DOI:** 10.3389/fpsyt.2025.1600212

**Published:** 2025-07-17

**Authors:** Song Bai, Miao Li, Shouying Tang, Suran Wan, Rong Wu, Lijun Chen, Fang Wang, Shan Liu

**Affiliations:** ^1^ Research Center for Green Chemistry and Ecological Environment Technology, Guizhou Industry Polytechnic College, Guiyang, China; ^2^ State Key Laboratory Breeding Base of Green Pesticide and Agricultural Bioengineering, Key Laboratory of Green Pesticide and Agricultural Bioengineering, Ministry of Education, Guizhou University, Guiyang, China; ^3^ Drug Identification Center, Guiyang Municipal Public Security Bureau, Guiyang, China

**Keywords:** drug substitutes, benefits, risks, analysis of technical bottlenecks, management strategies

## Abstract

The abuse of new psychoactive substances and narcotic drugs as drug substitutes poses a serious threat to public health and safety. This study outlines the effective use and negative abuse of drug substitutes and outlines associated criminal behaviors to help readers understand the potential risks of such substances. By summarizing the technical bottlenecks in the analysis and detection of criminal cases, this study highlights their impact on case discrimination, which in turn can limit or even render policy implementation ineffective. Finally, based on database analysis and the current regulatory system, flexible control strategies were proposed to address the constantly emerging “new” substances, providing a reference for relevant control guidelines to be formulated.

## 1 Introduction

With the rapid development of social economy, drug abuse has become an increasingly prominent issue posing a serious threat to social security and the physical and mental health of individuals. In recent years, the scale of drug supply, consumption, and abuse has continued to decline due to factors such as zero tolerance and high-pressure crackdowns on drug-related crimes by the international community. However, drug substitutes abuse has gradually increased, and new dynamics and trends have emerged in the field internationally. Drug substitutes are substances with effects similar to those of the original addictive substance that are sought by drug users for convenience and to satisfy their addiction due to an imbalance between supply and demand. These substances offer users convenience and a means to satisfy their addiction. Drug substitutes can be divided into the following categories according to their chemical composition, abuse methods, and effects: synthetic substitutes, natural plant substitutes, drug abuse substitutes, and new psychoactive substances. These substitutes often have addictive properties and can simulate or enhance the effects of traditional drugs in certain aspects while avoiding drug legislation (1–3). These compounds are popular worldwide because of their ease of purchase, lack of legal regulations, and incomplete safety assessment (4).

However, experimental, clinical, and epidemiological studies have shown that certain drug substitutes may cause chronic or acute health problems and pose unpredictable risks to human health (3–8). Abuse of drug substitutes by drug users can easily lead to mental abnormalities, hallucinations, or manic symptoms, posing a risk of accidents and even nonfatal and fatal poisoning hazards (5, 9, 10). Furthermore, in recent years, there has been a surge in the number of offenses involving drug substitutes, such as illegal manufacturing and distribution (11), involvement in narcotic-related sexual assaults (12, 13), accidental or intentional intoxication (6, 14–16), and recreational drugs abuse (16–19). However, in forensic and law enforcement departments, unlisted or newly emerging drug substitutes are excluded from standard drug screening protocols, and these may go undetected. Therefore, detecting new drugs in clinical and forensic setting is a complex task since traditional detection methods, such as mass spectrometry and chromatography, are reliant on known standard database that are difficult to update in real time. Simultaneously, other challenges such as sample complexity and matrix interference, trace detection and sensitivity limitations, and limitations of on-site rapid detection further complicate the analysis of certain drug substitutes. faces multiple challenges in the detection process. These analytical challenges are scientific in nature and directly affect the law enforcement, judicial system, and public health departments in their goal of prevention and control.

Drug traffickers use chemical modifications to synthesize alternative drugs and constantly challenge regulatory systems (such as the United States Federal Analogues Act) that are based primarily on chemical formulas. This force countries to explore more flexible policies to balance control accuracy with the need for quick response. In 1986, the United States revised the “Analogues Control Act” to explicitly state that substances sharing a parent structure or pharmacological similarity with a scheduled drug should be classified as a controlled analog (20). The purpose of this bill was to combat “underground scientists” who chemically modify the structure of listed substances, setting a precedent for the “skeletons control”. Subsequently, the UK followed suit by enacting the “Psychotropic Substances Act” in 2016, which established a parallel legislative response to address the rapid growth and increasing abuse of new substances. The “China Drug Situation Report 2019” (21) first proposed the concept of drug substitutes and mentioned their abuse in subsequent years. However, there was no mention of an explicit proposition of appropriate management policies for drug substitutes. These ambiguities in the formulation of laws and policies may affect case assessment and weaken the power of the law. Therefore, China may achieve collaborative innovation through the “China’s plan” and through international experience via “selective reference + localization transformation”. To break the vicious cycle of “ineffective control → accelerated diffusion” and build a more resilient drug control system.

Drug substitute abuse is becoming an increasingly serious issue and represents a key area for breaking the vicious circle of “regulation - variation - reregulation.” However, current research on drug substitutes is limited, lacking systematic understanding and in-depth research. This study provides an overview of the current trends and developments related to drug substitutes from several perspectives: explaining their pros and cons (knowing), analyzing their criminal effects (understanding), examining analytical detection methods (mastering), and prevention and control strategies (prevention). This study provides a valuable reference for drug abuse prevention and control efforts in China and contributes to the promotion of social harmony and stable development.

## 2 Duality of drug substitutes - benefits

Psychoactive substances or psychotropic drugs are chemical substances that affect human function as well as mental and behavioral states (22). Certain psychotropic substances have been reported to be relatively safe, inexpensive, readily available, of consistent quality (23), and highly effective (17). These characteristics highlight their potential antitoxic nature and value as alternative substances. Prior study findings have demonstrated that methadone or buprenorphine can be used as maintenance therapy to reduce mortality (24, 25); acetylfentanyl and butyrylfentanyl can rapidly inhibit morphine withdrawal symptoms in rhesus monkeys, with butyrylfentanyl demonstrating a morphine replacement effect (26); esketamine, the right-handed isomer of ketamine, with strong analgesic effects, a short awakening time, and weak inhibition of respiration and circulation, is widely used in the clinic (27, 28); oxycodone is used medically as an opioid analgesic and has the advantages of oral bioavailability, rapid onset of action, high efficacy in visceral pain, and fewer hallucinations (29, 30); etomidate compounds are used clinically for hypnotic sedation and anesthesia with rapid drug onset, rapid awakening, minimal respiratory and cardiovascular effects and reduced risk of hypotension (31, 32). In addition to this, some of the other drug substitutes with positive effects are shown in **Table 1**.

**Table 1:** Some drug substitutes with high detection frequency at present.

Legal status	Drug substitutes	Substitutes	Positive influence	Effect	Negative influence	Associated risks
Listed as a drug	Methadone (24, 25)	Herion	Medical field—opioid substitution therapies.	The drug is used with slow metabolism and elimination characteristics to avoid withdrawal symptoms in patients.	Entertainment abuse	①High risk of fatal respiratory depression. ②Cognitive decline ③Male sexual dysfunction ④Rapid increase in body temperature/blood pressure and blood drug levels.
	Buprenorphine (24)	Herion	Medical field—opioid substitution therapies.	The drug is used with slow metabolism and elimination characteristics to avoid withdrawal symptoms in patients.	Entertainment abuse	①High risk of fatal respiratory depression. ②Cognitive decline ③Male sexual dysfunction
	Esketamine(28)	ketamine	Medical field: ① Anti-inflammatory and pro-inflammatory effects. ② Analgesic and anesthetic effects.	①The awakening time is short. ②The respiratory and circulatory inhibitory effects are weak. ③Strong analgesic effect. ④High clearance rate in the body. ⑤Stress and inflammatory reactions, as well as adverse reactions, are relatively mild.	Entertainment abuse	①Elevated heart rate and blood pressure. ②Patients with reduced sympathetic nervous activity may experience decreased blood pressure and myocardial contractility.
	Oxycodone (29, 30)	Herion	Medical field—analgesics (for acute pain).	①The treatment of moderate to severe pain has a fast onset and high efficacy for visceral pain. ②Minimal impact on fetuses and newborns, can reduce postoperative complications. ③High oral biological utilization. ④Less histamine release, less hallucinations.	Tampered and misused	Common adverse effects: ①gastrointestinal disorders such as nausea, vomiting, or dyspepsia, ②nervous system disorders such as dizziness, headache, and somnolence.
	Etomidate (31, 32)	propofol	Medical field—hypnosis sedation and clinical anesthesia.	①The drug takes effect quickly. ②Awake quickly. ③Minimal impact on respiration and cardiovascular health. ④Reduced the risk of low blood pressure.	①Entertainment abuse ②Drug-assisted crime	①Paranoia and irritability ②Produce euphoria ③Addiction ④Cognitive impairment ⑤Brain damage ⑥Respiratory depression ⑦Potentially fatal injuries
	Metomidate (31, 32)	Etomidate and propofol	Veterinary field—sedative hypnotics.	①The drug takes effect quickly. ②Awake quickly. ③Minimal impact on respiration and cardiovascular health. ④Reduced the risk of low blood pressure.	①Entertainment abuse ②Drug-assisted crime	①Paranoia and irritability. ②Produce euphoria. ③Addiction. ④Cognitive impairment. ⑤Brain damage. ⑥Respiratory depression.
	Metonitazene (33, 34)	—	Medical field—emerging potent synthetic opioid.	Significant analgesic effect	①Entertainment abuse ②Drug-assisted crime	①common adverse effects: miosis, fatigue, reduced consciousness, nausea, vomiting, cyanosis, and respiratory depression. ②Potentially fatal injuries
	Etizolam (35)	Diazepam	Medical field—anxiolytic prescribed.	①Good biological utilization. ②Good anti-anxiety effect with gradual enhancement. ③No effect on cognitive function and psychomotor performance at therapeutic doses. ④Improvement in depressive symptoms.	①Non prescription use or non-medical use (illegal manufacturing and distribution, etc.) ②Entertainment abuse	①When used in high doses: central nervous system depression, slurred speech, severe sedation and unconsciousness ②When used in combination with other drugs: similar epilepsy, blepharospasm, and long-term myocardial toxicity occur ③Potentially fatal injuries
	2-oxo-PCE (36)	Ketamine and phencyclidine	—	2-oxo-PCE caused similar dissociative and hallucinogenic effects as ketamine, but with more prominent stimulant properties.	Entertainment abuse	①The main clinical symptoms after poisoning include consciousness disorders, blurred consciousness, abnormal behavior, hypertension, tachycardia, and convulsions. ②Potentially fatal injuries
	Propoxate and isopropoxate (37, 38)	Etomidate	—	The drug has induced anesthesia and sedation pharmacology: rapid onset of action, short duration of maintenance and high pharmacological activity.	Entertainment abuse	①Abuse can lead to symptoms such as severe immune decline, reduced vision, and decreased memory ②Long-term high intake: cause paranoia, anxiety, panic, victimization paranoia and other mental problems, triggering self-mutilation, injury or traffic accidents.
Not listed as a drug	Laughing gas (5, 39)	—	①Medical field—narcotic. ②Industrial field—dopants and propellants.	①The action time is relatively short. ②The mode of action will not affect the patient’s lung function and circulatory function. ③The functions of the digestive and excretory systems are not affected.	①Entertainment abuse ②Drug-assisted crime	①Mild: transient dizziness, disorientation, loss of balance, impaired memory and cognition, leg weakness, etc. ②Addiction can lead to various diseases, and in severe cases, it may cause mental disorders. ③Potentially fatal injuries.
	Pregabalin (40)	—	Medical field: ①Antiepileptic drugs. ②Treatment of generalized anxiety disorder and neuropathic pain ③Properties of mood regulation. ④Treatment of hypnosis-dependent insomnia. ⑤Benzodiazepine withdrawal. ⑥Treatment of alcohol dependence.	This drug can ①Attenuate the conditioned place preference effect caused by morphine. ②Reduce ethanol intake. ③Alleviate opioid withdrawal symptoms and dependence development. ④Enhance analgesic efficacy.	①Non prescription use or non-medical use (illegal manufacturing and distribution, etc.) ②Entertainment abuse and misuse. ③Experimental use of higher doses or modified forms of drug delivery.	①Joyful pleasure. ②Drunk feeling. ③Dizziness. ④It has an enhancing effect on other psychotropic drugs or poses an independent risk of abuse.
	Tiletamine (12, 41)	Ketamine	Veterinary field—compound anesthesia mixture.	①Induces a significant decrease in intraocular pressure elevation. ②Achieve muscle relaxation effect.	①Entertainment abuse ②Drug-assisted crime	①Decreased body temperature. ②Prolonged duration of nerve injury. ③Extended recovery time. ④Abuse addiction. ⑤Tachycardia.
	Cyclopropyl methoxycarbonyl metomidate (ABP-700) (42–45)	Etomidate	Medical field: ①Novel intravenous anesthetics. ②Dose-dependent hypnotic effect.	①Low impact on respiratory and cardiovascular functions. ②Does not cause severe hypotension, severe respiratory depression, or adrenal cortex depression. ③Does not increase plasma cytokine concentrations or mortality.	Entertainment abuse.	①Involuntary muscle movements with hemodynamic disturbances. ②Transient and self limiting upper respiratory tract obstruction. ③Epileptic seizures. ④Shortness of breath.
	Dextromethorphan (46, 47)	Herion	Medical field—cold and cough medicine.	The drug is less likely to produce opioid effects such as analgesia, central nervous system depression and respiratory depression.	Entertainment abuse.	①The practice of ingesting doses above those recommended, may produce a toxidrome of delirium with agitation, paranoia, and hallucinations. ②Other signs and symptoms may include tachycardia, hypertension, chest pain, drowsiness, dilated pupils, agitation, ataxia, dizziness, mental confusion, slurred speech, vomiting, tremors, headache, fainting, and epileptic seizures.
	1,4-butanediol (48, 49)	—	①Chemical field—chemical raw materials and solvents, etc. ②Plastic field—production of plastic raw materials.	①Efficient chemical reactivity. ②Lower toxicity. ③More environmentally friendly.	①Entertainment abuse. ②Drug-assisted crime. ③Chemical behavior.	①Transient neurological deficits. ②Longer period of loss of consciousness. ③Characteristics of alcohol intoxication.
	ADB-BUTINACA (50, 51)	Cannabis sativa	—	—	Entertainment abuse.	①Decreased level of consciousness. ②Respiratory and/or metabolic acidosis. ③Epileptic seizures. ④Blurred consciousness and hallucinations. ⑤Inducing cardiac dysfunction and possibly neurologic abnormalities.
	MDMB-4en-PINACA (52–54)	Cannabis sativa	—	—	Entertainment abuse.	①The mental symptoms are paranoia and anxiety. ②The digestive system symptoms are nausea and vomiting. ③Neurological symptoms include headaches, hallucinations, dilated pupils, amnesia, and epileptic seizures.
	The following “new” compounds have been discovered in recent years and have no information other than their chemical formulas and drug types.						
	Compound	Drug type	Structural formula	Compound	Drug type	Structural formula
	Protodesnitazene	Nitazenes	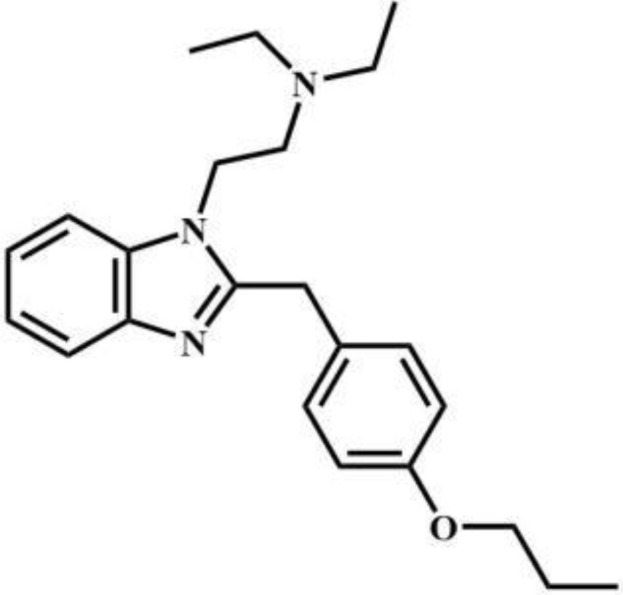	MET	Tryptophans	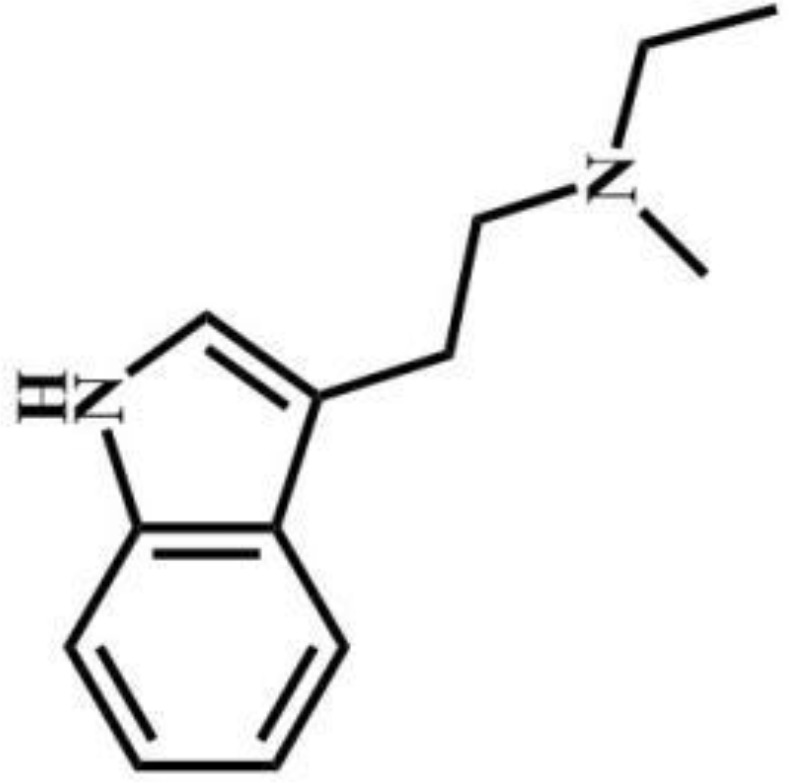
	2-FXPr	Phencyclidines	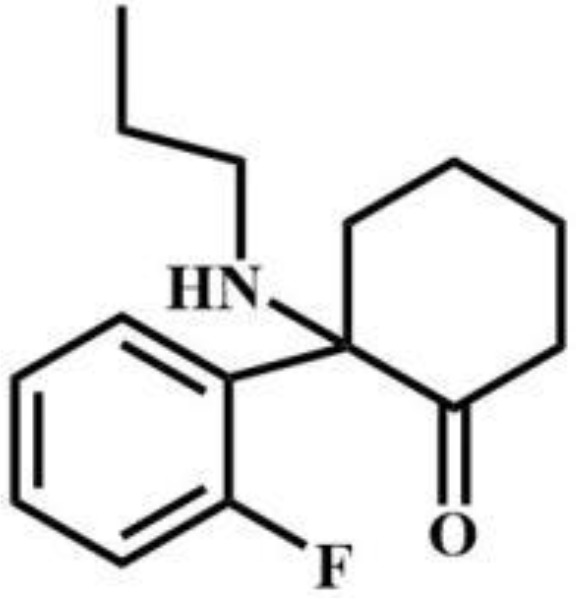	Methyldonazepam	Benzodiazepines	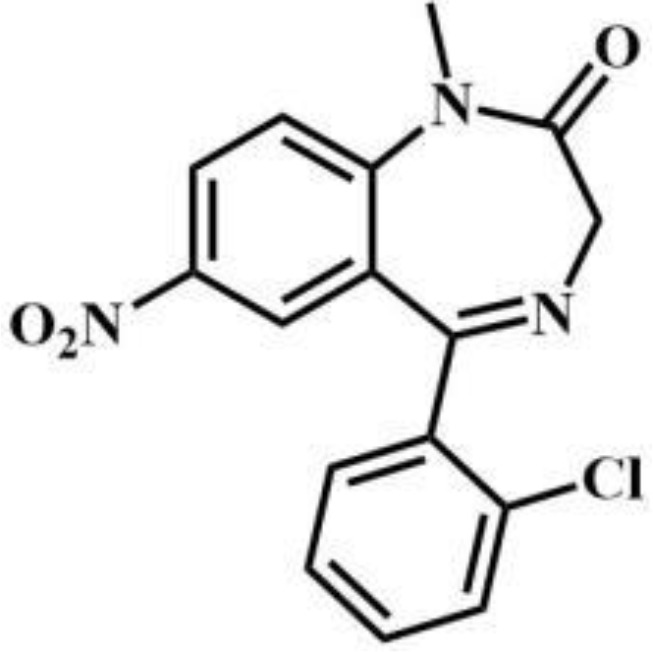
	Isobutylnitrite	Others	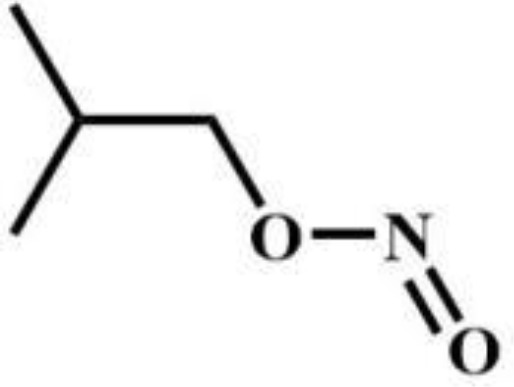	AB-MDMSBA	Synthetic cannabinoids	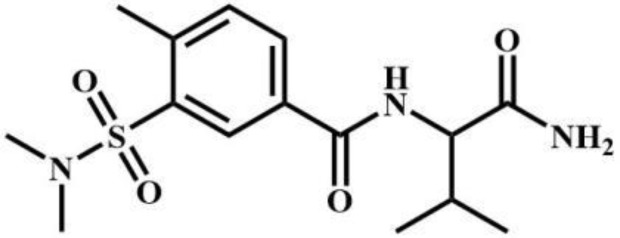
	sec-butomidate	Benzodiazepines	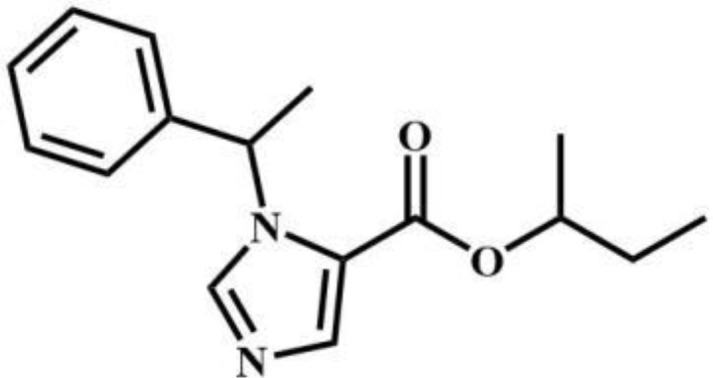	4F-etomidate	Benzodiazepines	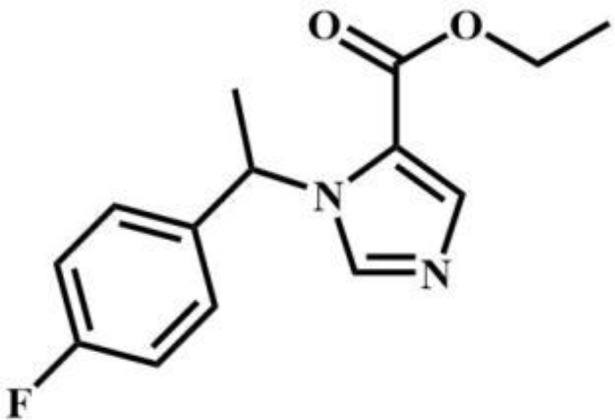
	butomidate	Benzodiazepines	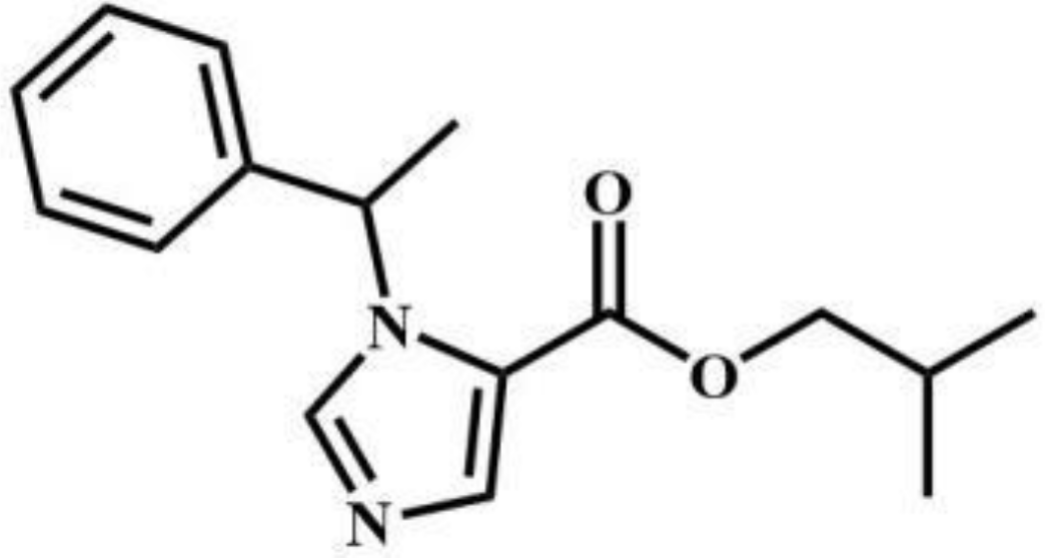	2, 6-dichloro-3fluoro-etomidate	Benzodiazepines	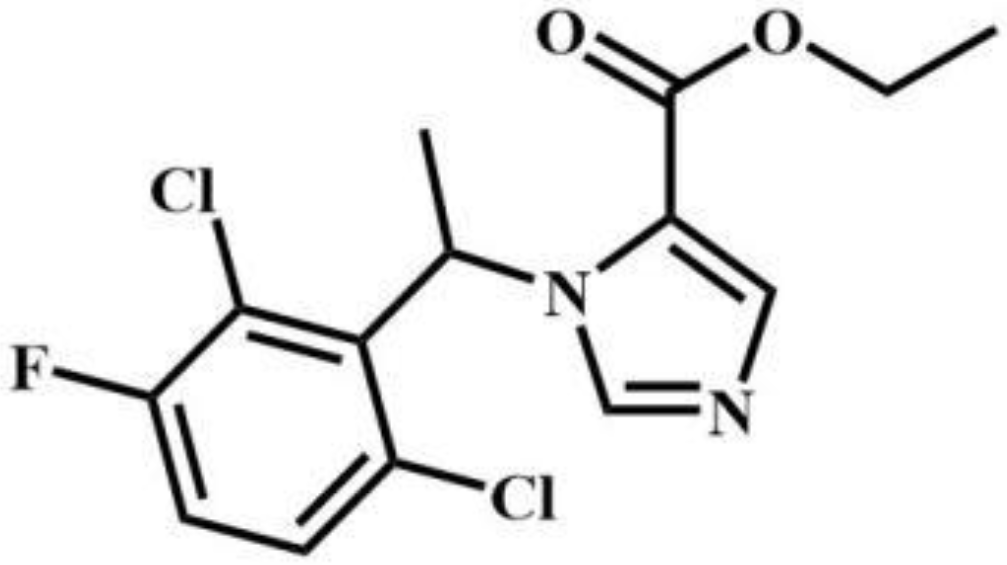
	CF_2_-etomidate	Benzodiazepines	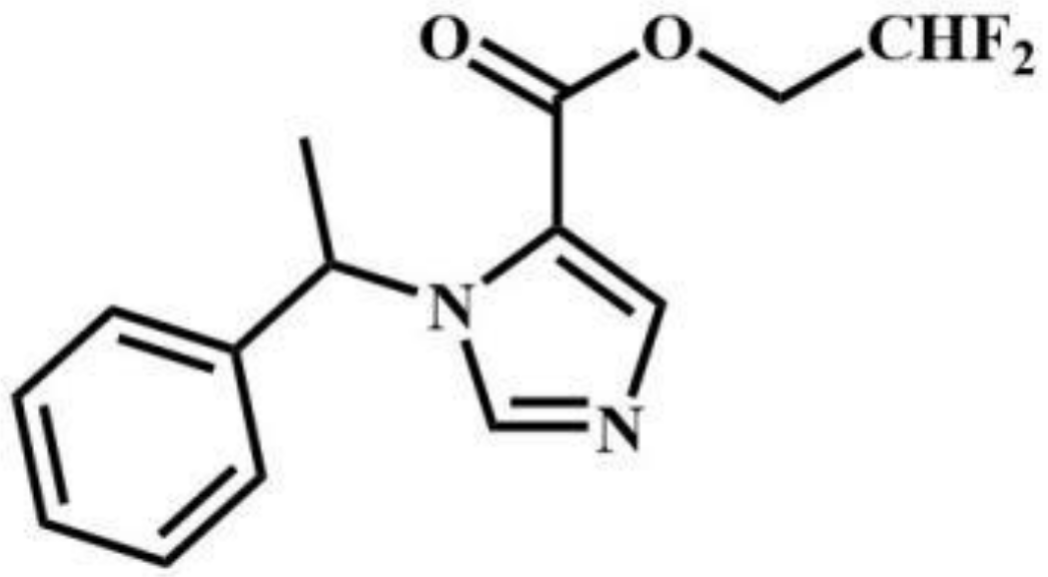	CF_3_-propoxate	Benzodiazepines	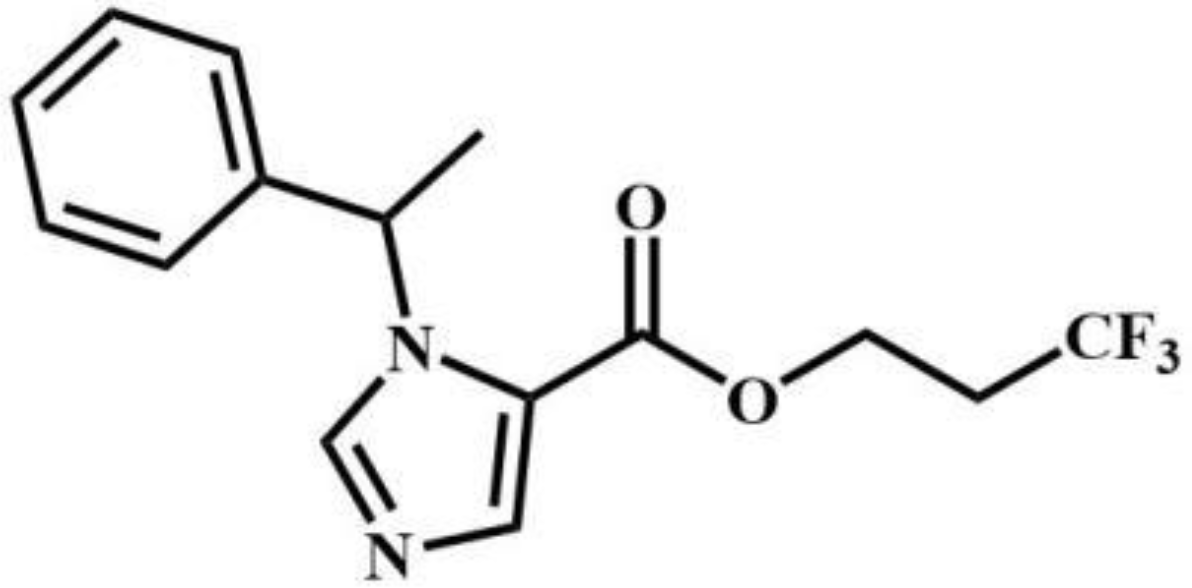
	CF_3_-etomidate	Benzodiazepines	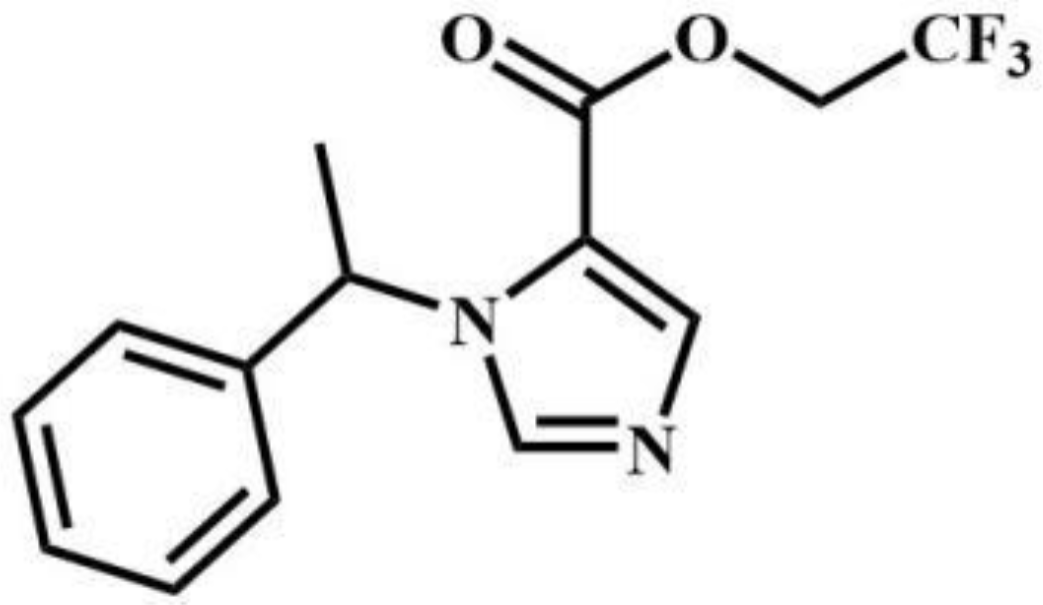			

“—” indicates unknown or not yet discovered.

Certain substances may have antitoxicity and alternative therapeutic properties. However, several experimental, clinical, and epidemiological studies have shown that most drug substitutes are associated with certain complications, including functional disorders, cognitive loss, multiorgan damage, poisoning, and high mortality rates (9, 10). Certain piperazine derivatives, with specific functional groups or substituents are used as anticancer drugs and exhibit good antitumor activity and low cytotoxicity in normal human liver cells, besides significantly inducing apoptosis, making them effective in an anticancer role (55, 56). As antipsychotic drugs too, piperazine derivatives exhibit strong activity to certain receptors. These antipsychotic effects are greatly enhanced when different heterocyclic groups are attached to the core piperazine structure or its rings (57, 58). However, piperazine analogs pose risks to humans as well such as life-threatening serotonin syndrome, hepatotoxicity, neurotoxicity, psychopathology, and potential abuse (4, 8, 59). “Laughing gas” is used medically as an anaesthetic (5), appreciated for its anxiolytic effects, and serves as an effective dopant in many industries (39). The industrial application of laughing gas constitutes a remarkable part of the national economy. However, its side effects include dizziness, disorientation, loss of balance, impaired memory and cognition, and leg weakness (5). Addiction to laughing gas can lead to various diseases, and, in severe cases, mental disorders and life-threatening complications.

The effects of drug substitutes on the body depend on single or multidrug intoxication (60, 61), route of administration (62), the person’s medical history (63), overdose, and medication purposes (64–66). To mitigate adverse events, it is essential to understand the pharmacological properties and common uses of drug substitutes prior to application, as well as ensure strict supervision and standardized guidelines are being followed during use. In summary, based on the progress in basic and clinical medical research, it is necessary to transform societal knowledge, attitudes and behaviors toward various types of drug substitutes. A comprehensive understanding of the relationship between drug substitutes and individuals, society, and countries should be considered at different levels and in varied perspectives. Strengthening the management of drug substitutes by updating the drug catalog can ensure their safe and effective use while maximizing their potential benefits.

## 3 Duality of drug substitutes - risks

Drug substitutes have been of use in the material sciences world for a long time and were originally intended to promote the development of this field (24–33). However, due to its traditional drug like effects, easy access, and gray areas in legal regulation, this type of substance has gradually become the focus of attention for drug traffickers. In recent years, drug substitutes have been found in suspected drug-related crimes; however, the degree of abuse is relatively mild (67, 68) and involves substances such as benzodiazepines, hypnotics, sedatives, and anesthetics. Initially, the emergence of these substitutes was likely driven by a lack of awareness of their potential harm and a desire for the effects these psychoactive substances provided. With changes in society and the strengthening of drug control measures, the supply of these addictive substances became insufficient and expensive. This led drug traffickers to seek drug substitutes with similar effects for sale or to commit crimes (11–19).

Drug substitutes are not confined to a closed range, and any addictive substance has the potential to become a substitute for abuse. This highlights the diversity and complexity of criminal targets, the interconnected and varied forms of criminal organization forms and the globalization and internationalization of crime-related regions. According to the United Nations Office on Drugs and Crime, a high proportion of new psychoactive substances-related deaths reported in 2019 were related to kratom, which has opioid and stimulant properties and contains pharmacologically active alkaloids. Between 2017 and 2019, the US Toxicology Portal reported 47 cases of kratom infection. Of these, 29 individuals died, 16 consumed it under the influence of alcohol, 1 was sexually assaulted, and 1 incident occurred in public places (69). Studies have indicated that xylazine (a veterinary drug) can also be used as a drug substitute in humans. Xylazine misuse has been linked to both accidental and intentional poisoning (6, 14, 15), recreational use, and use as a recreational drug admixture (18, 19). When administered to humans, it may cause central nervous system and respiratory depression, bradycardia, hypotension, and death. In addition, certain drug substitutes listed as illicit drugs are used for other illicit incidents, including drug trafficking (11), narcotic-related crimes (12, 13), lethal injections (15), recreational abuse (16, 17), and use as sedatives for animals reared for food and as stimulants for sports animals (19, 70). In addition, according to the results of the analysis of biological samples (hair, urine, and tobacco oil) conducted by the Drug Identification Center of the Guiyang Municipal Public Security Bureau in recent years, it was found that Pregabalin (41), ABP-700 (42–45), Dextromethorphan (46, 47), ADB-BUTINACA (50, 51), MDMB-4en-PINACA (52–54) and protodesnitazene were not included in the prohibited list, and the detection rate continued to increase. The relevant information on these drug substitutes is provided in **Table 1**.

Regarding the constantly emerging “new” substances and “new” crimes, White et al. (71) and Goldstein et al. (72) argue that drugs induce psychoactive effects that reduce natural inhibitions, thereby altering the drug user’s perception of the risks and consequences of crime. From an economic standpoint, drug users commit crimes as a means to finance their drug use. This trade in illegal drugs constitutes a crime and can lead to other criminal activities. In other words, if drug substitute usage lead to criminal behavior, then this criminal behavior creates further demand, sustaining the market for such substances. When existing laboratory analysis techniques fail to detect “new” drug substitutes circulating in the market or when legal loopholes exist, traditional regulatory measures become ineffective. This highlights the urgent need to develop cutting-edge analysis methods capable of rapidly identifying these substances, tracing their criminal use, and supporting judicial efforts. Such innovations are essential for providing scientific evidence that can disrupt the chain of the criminal behavior associated with drug substitute crimes.

## 4 Technical bottlenecks in the analysis of drug substitutes

According to the World Drug Report 2023 published by the United Nations Office on Drugs and Crime, new psychoactive substances are being continuously added globally in recent years, with only a few identified in laboratories within a short period (73). This lag in detection technology can undermine the reliability of scientific evidence, delay legal responses, exacerbate resource allocation imbalances, and, ultimately, result in limited or ineffective policy implementation. Laboratory testing currently focuses on the challenges of samples, instruments, and standards.

Appropriate biological samples are fundamental to the scientific and legal effectiveness of drug analysis. Such robust samples enable qualitative identification of substances present in drug users and provide targeted, highly accurate quantitative information about one or multiple drug substitutes (74). For general drug abuse cases, blood, urine, hair, and saliva are commonly used samples as they reduce the impact on the examinee In the case of collection of samples following death, peripheral blood, cardiac blood, spleen tissue, injection site tissue, and stomach tissue as well as the contents of the stomach are collected to provide a more comprehensive analysis of the type, duration, and form of substance abuse in the deceased’s body (15, 75). If necessary to determine whether alcohol has been consumed, ocular vitreous fluid should be collected simultaneously (75). Due to the high rates of sexual crimes worldwide, Amanda et al. (76) collected semen samples from crime scenes or victims as biological samples for testing. This method overcomes the limitations of traditional testing and provides support for criminal investigations. In addition, with the further optimization of modern analytical tools, the choice of biological samples has become wider. As Drolet’s et al. research has shown, dried blood spot and dried urine strip methodologies are minimally invasive sample collection methods that allow for ease of sample collection at minimal cost (77). The selection of samples must be comprehensively evaluated and considered based on the purpose of detection, toxicokinetic characteristics, legality of sample collection, and suitability of technical methods.

From the perspective of production methods, most drug substitutes produce new substances with structures similar to those of the original substances by modifying certain groups in the chemical structure (78–85). When these drug substitutes and their metabolites have similar chemical structures to those of their prototype drugs, simple test plates can be used to test positivity; however, these can lead to false-positive results (11, 86). Therefore, the detection and analysis of drug substitutes with similar structures typically require high-precision instruments such as mass spectrometry and liquid chromatography. Tiletamine and telazol have structures similar to those of ketamine, which can easily lead to false-positive immunoassay results. In such cases, advanced testing methods can be used; such as the case where He et al. (12) and Quail et al. (17) used GC-QTOF-MS and gas chromatography-mass spectrometry for detection and provided accurate mass numbers post-analysis. Previous studies have shown that the detection of etomidate and/or etomidate acids in biological samples is indicative of drug abuse since etomidate acid is a common metabolite of etomidate and its analogs (31, 32). However, etomidate analogs currently available on the market are not listed in **Table 1**. Tang et al. (31, 32) used high-resolution liquid chromatography-mass spectrometry to analyze specific metabolites and track prototype compounds to avoid misjudgment of drug abuse cases. This provides a reference for analyzing these substances. However, the dependence on high-end equipment, regional capacity differences, and imbalanced allocation of mass spectrometry parameter setting resources among countries have led to marked differences in the technical level of forensic toxicology analysis among laboratories. At the same time, drug testing, analysis, and identification in various countries are mainly concentrated in the National Drug Laboratory and a few qualified appraisal institutions. Most research institutions with the necessary capabilities are currently unable to perform relevant analyses and identifications, hindering the rapid development of detection and analysis.

Laboratory analysis results are highly dependent on the availability of standards and databases. However, both are subject to delayed update cycles. The emergence of new alternatives to detection and re-listing follows three steps: submission of applications, risk assessment, and formal listing (87, 88). During this time lag, the market share of these drug substitutes expands sufficiently to support the next round of structural variation. Currently, only a few foreign companies can provide standard substances for newly identified substances, and these are often expensive and limited in variety; therefore, many laboratories are unable to obtain high-quality reference substances (86), possibly leading to the misclassification of legal drugs as substitutes and weakening of the public trust in antidrug policies. However, many substitutes are metabolized into unknown compounds within the human body, making it difficult to trace them back to the original substance using existing technologies or standards. In legal proceedings, if the court requires clear evidence linking drug ingestion to individual behavior but the metabolites do not correspond to the original substance, the case may be dismissed due to insufficient evidence. This may lead to a cycle of “detection lag or insufficient evidence → regulatory delay → market diffusion → technology catch-up”.

Therefore, it is necessary to continuously expand the scope of drug substitute testing and improve, update, and optimize the screening and analysis processes internally. When the iteration speed of drug substitutes exceeds the cognitive boundary of traditional detection technologies, innovation in analytical methods is no longer merely a technical proposition of the laboratory.

## 5 Loopholes in the current control system and the proposal of new control ideas

The “structural escape” strategy designed by drug traffickers—exploiting delays in mass spectrometry databases—constantly challenges regulatory systems that rely on chemical formulas as the control. This has forced policymakers to find a new balance between “precise legislation” and “flexible control”. When a new substitute emerges in the drug market, legislators request a risk assessment by the World Health Organization’s drug expert committee on the relevant substance (87). If the assessment shows that the substance pose a risks that outweighs its potential therapeutic value, it may be added to the International Drug Control Convention for Regulation. For a period of time, legal restrictions on the sale of gamma hydroxybutyric acid in the UK have actually led to an increase in the use of the drug GBL (89, 90), and subsequent legislation targeting GBL contributed to the emergence of substitutes with higher side effects. The prohibition of methamphetamine has led to the rapid and widespread popularity of its legal substitutes such as BZP (78) and U-47700 (79). After a period of time, both were listed as controlled drugs, leading to the rapid emergence of D2PM and mephedrone (91) as substitutes of unknown toxicity. Furthermore, substitutes for substances such as ketamine, heroin, and morphine, such as isoflurane (80), fentanyl (81), 4-fluoroamphetamine (82, 83), and cassia seed (84, 85), have been in widespread use for some time now. Subsequently, due to reports of poisoning and death from these alternative drugs, these alternatives were added to the list of controlled drugs.

However, when these harmful substances disappear from the drug market, alternative substances that cause greater harm may emerge (3, 92). This poses a new problem, as the user group knows little about these compounds and is almost unaware of the risks posed by these new drugs. This leads to the question of whether this is a good strategy to drive all drug substitutes to the market immediately. Another approach proposed by foreign policymakers to address this issue is to accept reality, formulate policies, tolerate substances with the least risk, and prevent user groups from shifting from one substance to another, thereby reducing the risk of substance use (93). While considering systematic solutions, it is good to create favorable policies that align with local culture, background, and politics (94).

Based on existing international and domestic management regulations and social development needs, this study proposes the following hypotheses. First, in light of China’s national conditions, a temporary control system should be established, drawing on the practices of the United States, United Kingdom, Russia, and other countries (95). When a new type of substance that poses a serious risk to public health emerges in the a country, it may be subjected to temporary scheduling for a period of 1–2 years, during which a risk assessment can be conducted before deciding whether to officially list it. At the same time, after the activity and effects of the substance have been assessed by a judicial appraisal agency or scientific researchers, the information currently available will be made public; therefore, the public can become aware of the substance and reduce its use to minimize the risks involved. Second, after formal listing, relevant departments should issue judicial interpretations to establish standards for prosecuting and convicting individuals involved in the manufacture and trafficking of such substances. Subsequently, an authoritative identification agency should be established at the national level; a library of standard substances and analytical charts should be established; analytical methods based on common laboratory instruments should be developed; and analytical methods for biological samples suitable for on-site law enforcement testing should be developed to enhance the level of awareness and detection capabilities. Finally, supervision of the production and distribution of drug substitutes should be increased. The distribution of synthetic raw materials for medicines is controlled, and enterprises that produce, process, and sell new active substances without obtaining registration certificates should be dealt with in accordance with the law. Strengthening internet monitoring, timely deletion of websites containing the content of selling and trafficking drug substitutes, and dealing with relevant websites is also of utmost importance. Regardless of the approach taken, it will need to change over time to deal with this dynamic market and the increasing number of innovative attempts to circumvent regulations.

To strengthen the control of drug substitutes, it is necessary to work simultaneously on aspects of prevention, monitoring, and enforcement. First, demand-side interventions should be carried out for high-risk groups based on the trend of abuse to eliminate the possibility of abuse on this side. Simultaneously, monitoring and testing should be strengthened to accurately determine the properties of suspicious substances. It is necessary to strengthen the fight against illegal production, transport, sale, and possession of these substances. Finally, it is important to understand the degree of control required to prevent criminals from shifting their attention to compounds with higher risks. In other words, the management of drug substitutes should be based on the premise of “necessity” and should be undertaken without affecting economic and social development to achieve a balance between “necessary management” and “legitimate use”.

## 6 Conclusion

This study revealed the duality of current drug substitutes, including the effective use of medical and industrial drugs and the negative effects of illegal abuse and crime. The continuous emergence of drug substitutes leads to criminal cases, presenting multiple challenges for forensic investigations and laboratory analysis. These analytical challenges complicate law enforcement efforts and policy responses, ultimately weakening public confidence in antidrug policies. We also analyzed the unintended spread of more harmful new alternatives following prohibition measures of the older substances and proposed solutions informed by current regulatory management and social development needs to help mitigate this. In summary, this study aimed to systematically understand drug substitutes to reduce the risk of exposure. Moreover, it provides an analysis of the current situation of drug substitutes for public security organizations, enabling them to better respond to changes in the drug environment and maximize the effectiveness of combating drug substitute–related crimes.
